# Characteristic Features of Ultrafine-Grained Ti-45 wt.% Nb Alloy under High Cycle Fatigue

**DOI:** 10.3390/ma14185365

**Published:** 2021-09-17

**Authors:** Aikol M. Mairambekova, Anna Y. Eroshenko, Vladimir A. Oborin, Mikhail V. Bannikov, Valentina V. Chebodaeva, Alena I. Terekhina, Oleg B. Naimark, Andrey I. Dmitriev, Yurii P. Sharkeev

**Affiliations:** 1 Institute of Strength Physics and Materials Science of SB RAS, 2/4, Akademicheskii pr., 634055 Tomsk, Russia; aikol@ispms.ru (A.M.M.); eroshenko@ispms.ru (A.Y.E.); vtina5@mail.ru (V.V.C.); sharkeev@ispms.ru (Y.P.S.); 2 Department of Solid Mechanics, Faculty of Physics and Engineering, National Research Tomsk State University, 36, Lenina pr., 634050 Tomsk, Russia; 3 Institute of Continuous Media Mechanics of UB RAS, 614013 Perm, Russia; oborin@icmm.ru (V.A.O.); mbannikov@icmm.ru (M.V.B.); terekhina.a@icmm.ru (A.I.T.); naimark@icmm.ru (O.B.N.); 4 Research School of High-Energy Physics, National Research Tomsk Polytechnic University, 30, Lenina pr., 634050 Tomsk, Russia

**Keywords:** Ti-45 wt.% Nb alloy, ultrafine-grained structure, fatigue testing, surface morphology, Hurst exponent, infrared thermography method

## Abstract

The paper presents the results of fatigue-testing ultrafine-grained and coarse-grained Ti-45 wt.% Nb alloy samples under very high cycle fatigue (gigacycle regime), with the stress ratio R = −1. The ultrafine-grained (UFG) structure in the investigated alloy was formed by the two-stage SPD method, which included multidirectional forging (abc–forging) and multipass rolling in grooved rollers, with further recrystallization annealing. The UFG structure of the Ti-45 wt.% Nb alloy samples increased the fatigue limit under the high-cycle fatigue conditions up to 1.5 times compared with that of the coarse-grained (CG) samples. The infrared thermography method was applied to investigate the evolution of temperature fields in the samples under cyclic loading. Based on numerical morphology analysis, the scale invariance (the Hurst exponent) and qualitative differences for UFG and CG structures were determined. The latter resulted from the initiation and propagation of fatigue cracks in both ultra-fine grained and coarse-grained alloy samples under very high-cycle fatigue loading.

## 1. Introduction

The prediction of fatigue strength and the development of further upgrading methods to improve the structure of materials in engineering and medical applications are the current most popular research directions, embracing applications in different areas of materials science. Due to its high specific strength, improved fatigue life, corrosion resistance and biocompatibility, titanium and its alloys are among the most widely used materials in different industrial applications, including the production of medical implants. The most popular titanium-based materials are commercially pure titanium, Ti–6Al–4V, Ti–6Al–7Nb, Ti–6Al–2.5Fe, Ti-15Mo titanium alloys and others [1]. However, the production of medical implants requires materials without any toxic alloying elements, such as Al, V, Mo and others, which could negatively affect the organism [1,2,3,4]. In this respect, the most promising materials for medical applications are valve bioinert metals, such as Ti, Nb, Zr, Hf, Ta and others [5,6]. Bioinert metal alloys could be used for manufacturing carrier implants for artificial hip joints, bone plates and instruments for spinal and dental screws, which are subjected to cyclic loading [1,2,3,4,5,6,7].

One of the main disadvantages of metallic implants that are used in medicine is the difference in the mechanical behavior of the bone tissue and implant materials, which result in the uneven distribution of deformations and mechanical stresses at the bone-implant interface, as well as in the increased risk of the breakdown at the rigid fixation of the implant to the bone. The elastic modulus of titanium and its alloys (above 100 GPa) is significantly higher than that of bone tissue, which ranges from 10 to 30 GPa [7,8]. In this case, the most promising choices are β–titanium alloys, which include non-toxic alloying elements and are characterized by a low elastic modulus. Ti-Nb alloy systems embracing 40–45 wt.% of niobium possess a rather low elastic modulus (50–60 GPa) [1,4]. In this range of Nb concentrations, the alloy is a mixture of α + β phases [8,9,10,11]. Under the conditions of mechanical and thermal loading, both balanced phase conversion and the formation of metastable phases originate in the Ti-Nb system. It should be noted that, as is similar to the stoichiometric Ti-Nb composition, the alloy has a high-temperature superconductive state [12].

It is well known that the different parts and structural units of medical implants are subjected to continuous cyclic loadings. Therefore, the development and production of high-strength alloys are of significant importance in improving implant fatigue life. The formation of bulk nanostructured (NS) and/or ultrafine-grained (UFG) structures in metals and alloys by different severe plastic deformation (SPD) methods, followed by thermo-mechanical treatment, profoundly improves their mechanical properties (yield strength, ultimate strength, fatigue strength, fatigue life, etc.) [13]. It has been reported by numerous researchers [14,15,16,17,18,19,20] that the formation of NS and/or UFG structures in metals significantly increases the fatigue limit under high cycle fatigue (more than 10^6^ cycles), and very high cycle fatigue (more than 10^9^ cycles). Several published investigations [19,20,21,22,23,24,25] have shown the influence of grain size on the fatigue strength of alloy samples with or without notches, subjected to different loading regimes (quasi-static and cyclic). It has been emphasized that the strength has been significantly improved in the case of the fine-grained structure. Due to the high sensitivity of titanium alloys to the presence of a notch, the surface morphology of the samples should be considered in order to predict the material fatigue strength and predetermine the material behavior at the early stages of crack initiation and propagation. It should be noted that the fatigue failure mechanisms of biocompatible low-modulus alloys, in particular the Ti-45 wt.% Nb alloy, when subjected to high cycle fatigue and very high cycle fatigue (gigacycle regimes) are poorly studied [23,24,25].

Traditional methods do not provide an assessment of the gigacycle fatigue life that has led to the emergence of new techniques. It is known that intensive heat release on the material surface, structural decomposition and the accumulation of structural defects originate during cyclic loading [26,27,28,29,30,31,32]. The high sensitivity of modern infrared cameras, coupled with non-contact temperature measuring systems, conditioned the application of the infrared (IR) thermography method. The method provides detailed information on the processes of the initiation and propagation of fatigue cracks, as well as energy conversion and accumulation during mechanical testing. Unfortunately, there is little information about the application of IR thermography for investigating the heat-generation processes in UFG metals during fatigue-testing [30,31,32,33]. Non-linear vibration analysis [34,35,36] is widely used to define the initiation and growth of fatigue cracks [37,38]. This method provides an in situ evaluation of material damage during testing. This is based on the anomalous enhancement of elastic properties, measured by the second harmonic oscillation amplitude of the free sample end. The issues in question are associated with the investigations of energy transition and accumulation behavior, as well as the damage mechanism during the high-cycle and gigacycle fatigue testing, but they remain uninvestigated to the present day and require detailed analysis, including those for Ti-45Nb alloys.

Another effective technique for studying the mechanisms of crack initiation during the gigacycle loading is based on the use of the previously established relationship between the evolution of an ensemble of defects during fatigue failure and the scale-invariant characteristics of the fracture surfaces [39,40]. The methods developed for the quantitative analysis of surface morphology, based on the calculation of the Hurst exponent [41], make it possible to identify the mechanisms and stages of fracture development under both high-cycle and gigacycle fatigue.

Thus, in this work, the effect of the structure of low-modulus Ti-45 wt.% Nb alloy on its fatigue behavior under high-cycle and gigacycle fatigue was studied and the specific damage mechanisms were determined, based on microstructure analysis, temperature evolution and surface morphology analysis.

## 2. Materials and Methods

**Figure 1:**
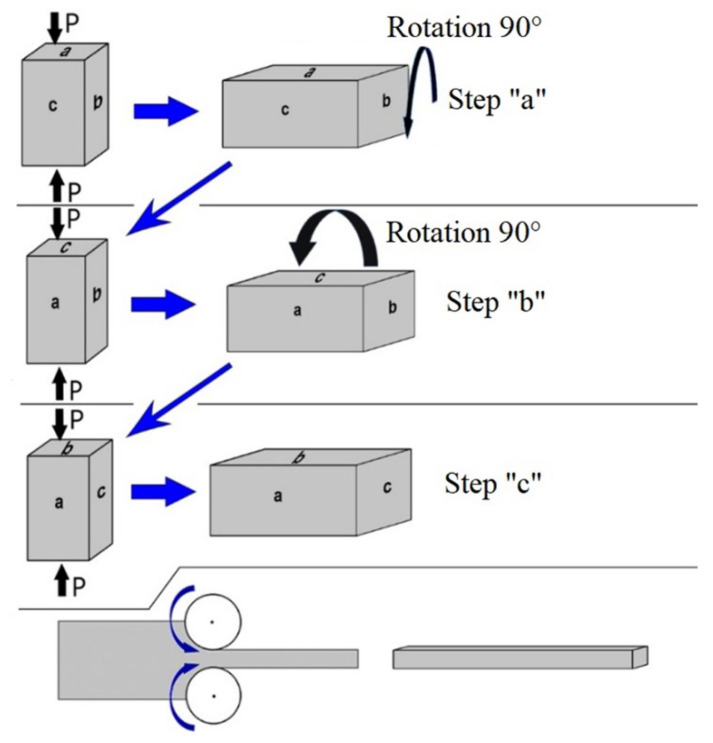
Multidirectional forging, including rotation of the deformation axis (abc-forging) and further rolling in grooved rollers.

The material used for this investigation was a bioinert Ti-45 wt.% Nb (Ti-45Nb) alloy in both coarse-grained (CG) and ultrafine-grained (UFG) states. According to the standard classification [42], the UFG state corresponds to a range of sizes of structural elements (grains, subgrains, and fragments) from 0.1 to 1 µm, and the CG state corresponds to a range of 10–100 µm. The chemical composition of the Ti-45Nb alloy is presented in Table 1. Ti-45Nb rods were produced in the Chepetsky Mechanical Plant JSC (Glazov, Russia). The as-received rods were cut into billets (40 mm in length and 25 mm in diameter) for further molding and pressing, followed by plastic deformation. The UFG structure in the investigated alloy was formed by the two-stage SPD method, which included multidirectional forging (abc–forging) and multipass rolling in grooved rollers, with further recrystallization annealing [42,43,44]. The method of producing the Ti-45Nb alloy billets is presented in Figure 1.

At the first stage, the deformation of the billets involved three pressing cycles. Each cycle included one-step pressing at a given temperature. From one pressing cycle to the next, the temperature during billet-pressing was decreased step-by-step by 50 °C, in the range of 500–400 °C. After each pressing cycle, the billet was rotated by 90° around its axis, perpendicular to the previous pressing step. The relative deformation of the billet in each pressing cycle was 40–50%. It should be noted that the three-cycle pressing method was already used to obtain the UFG state in the alloy. However, the microstructure after the pressing was non-uniform. Therefore, the second stage of the SPD was applied to obtain a homogeneous structure.

At the second stage, the Ti-45Nb alloy billets were deformed through multi-rolling in grooved rollers at room temperature. The accumulated deformation during rolling was 75%. The rolling in the grooved rollers enabled the formation of a homogenous structure in the bulk of the billet. Square bars of (8 × 8 × 200) mm^3^ were produced after rolling. The combination of abc-pressing with rolling provided the formation of a finer-dispersed UFG structure, compared with the structure formed after the abc-pressing. To reduce the internal stress and increase elasticity, the produced Ti-45Nb alloy rods were annealed at 350 °C [42,44] for 1 h in an argon atmosphere and cooled in the furnace. Annealing at 350 °C did not change the structure-state of the materials but it did increase the elastic limit. First, a coarse-grained (CG) state in Ti-45Nb was formed, then UFG billets were obtained after recrystallization annealing in a vacuum at 800 °C for one hour. Recrystallization annealing made it possible to obtain equilibrium in the CG structure. The average grain size in the CG state was 45 μm, while for the UFG state it was 0.20 μm.

The sample microstructure and phase composition were investigated by optical microscopy (Carl Zeiss Axio Observer, Zeiss, Jena, Germany) and transmission electron microscopy (JEOL JEM 2100 microscope, Akishima, Japan). An average-structure element (grains, subgrains, fragments) size was calculated by the “linear secant” method [45].

This research involved two experiments. The first experiment was conducted under very high cycle fatigue (gigacycle regime) with air cooling of the sample. The second experiment was performed on the process of heat release without air cooling at room temperature, by applying IR-thermography in the real-time mode.

**Figure 2:**
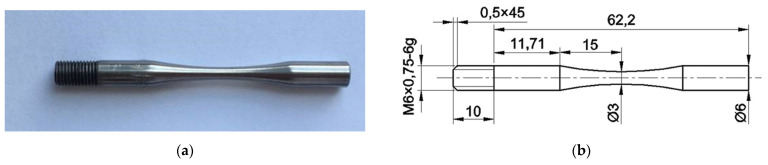
(**a**) Shape and (**b**) dimensions (in mm) of the Ti-45Nb alloy sample for fatigue-testing.

The hour-glass-shaped samples (Figure 2a) were produced from coarse-grained and ultrafine-grained Ti-45Nb alloy rods, using a turn-milling machine (GLS1500LY, Goodway Machine Corp., Taichung City, Taiwan). The sample dimensions (Figure 2b) were calculated according to the analytic formulae [18], based on the material density and its dynamic Young’s modulus and responding to the conditions of standing ultrasonic waves and the resonance of the testing system. To decrease the possible crack initiation on the material surface due to strain concentrations during testing, the sample surfaces were polished to a *Ra* roughness of less than 0.6 µm.

**Figure 3:**
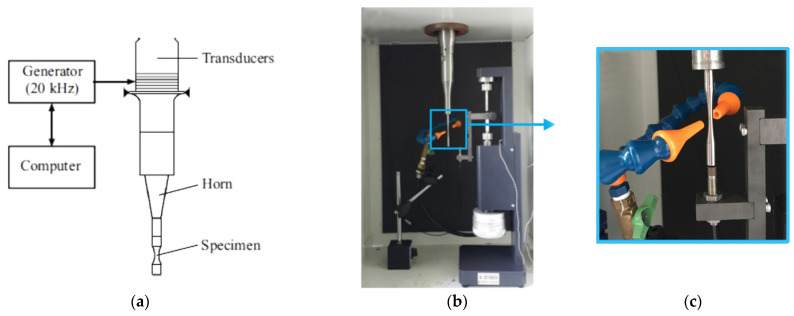
(**a**) Schematic and (**b**) overview of the ultrasonic resonance loading machine Shimadzu USF–2000 under cycle testing; (**c**) air cooling system [48].

Very high cycle fatigue-testing was conducted using an ultrasonic resonance loading machine, Shimadzu USF–2000, (Kyoto, Japan, Figure 3a,b), by the push-pull method, with a frequency of 20 kHz and a stress ratio R (σ_min_/σ_max_) = −1. The system itself was in the resonance mode, whereas the maximum amplitude of standing waves (mechanical stress) was in the working part of the sample; while the maximum of the displacement wave is at the sample ends [18,46,47,48,49]. Air cooling was applied (Figure 3c) to prevent violation of the resonance mode, due to changing sample elastic properties arising from its heating to 200–400 °C during the fatigue-testing.

The Rizitano-Luong method [26,27,28,29,30,31] was applied to estimate Ti-45Nb alloy continuous fatigue life, based on short-term experiments. This method of determining the fatigue limit is based on extreme heating of the material during cycle-testing under loading amplitudes, close to the fatigue limit. The application of IR, coupled with the testing machine, significantly speeds up the process of fatigue limit determination. In this case, the testing was conducted in the gigacycle fatigue regime at a cycle frequency of 20 kHz and the stress ratio R = −1, excluding air-cooling at room temperature and applying IR thermography (Figure 3).

According to the gigacycle testing experiment data, the loading amplitude was selected as 80% of the fatigue limit for the CG and/or UFG Ti-45Nb alloys. The testing started with a loading amplitude of 100 MPa for the CG samples and 195 MPa for the UFG samples and was further increased step-by-step by 2–10 MPa. Maximal temperature changes on the Ti-45Nb alloy surface were determined in the working part of the sample. After achieving the specified cycle numbers and/or stabilizing the average temperature on the sample surface, the testing was stopped. Then, the loading amplitude was increased and the experiment was repeated. The stress amplitude that corresponded to a sharp increase in average temperature was identified with the fatigue limit [30].

The temperature distribution during the cycle testing was recorded as a digital thermograph obtained with an IR camera FLIR CEDIP Silver 450 M (Danderyd, Sweden) in the real-time mode [30,31]. The sample surface temperature was recorded within a frequency of 1600 Hz and a maximal spatial resolution of 2 × 10^−4^ m. The camera spectral range was 3–5 µm. The maximal frame dimension was 320 × 256 pixels. The camera sensitivity was not less than 25 mK at a sample temperature of 300 K. The data analysis and processing, and the visualization of temperature fields were accomplished using the Altair software [31]. To improve the visualization quality of the temperature fields, the sample surfaces were preliminarily covered with thin amorphous carbon layers. 

For a comparative analysis of the energy dissipation processes in the investigated Ti-45Nb alloy for two states, CC and UFG, lines of equal temperature (isolines) were drawn on IR images, reflecting the temperature distribution over the sample surface during testing. The determined temperature points were equal to or more than the temperature points on the isoline with respect to the given temperature. The measurements were conducted using the following parameters: a stress amplitude of 195 MPa and the heat zone limited by ~86 °C isoline. The dependence of the longitudinal heat zone on the number of fatigue cycles was plotted for the given temperatures and stress amplitudes.

The comparison analysis of fracture surface of Ti-45Nb alloy samples after gigacycle testing was conducted to investigate the fatigue failure behavior. Fracture surface morphology was examined with a scanning electronic microscope (SEM) (LEOEVO 50 Carl Zeiss AG, Oberkochen, Germany), coupled with an energy-dispersive spectroscopy detector (INCA Energy-250, Oxford Instruments, Tokyo, Japan). A series of samples, embracing surface cracks within the working part, was preliminarily cooled in liquid nitrogen.

The crack surface was examined by fractal analysis [47] to determine the conditions of correlated multiscale defect structure behavior associated with crack initiation and propagation. The surface crack patterns were investigated with an optical high-resolution interferometer–profiler (ZYGO New View 5010, Middlefield, CT, USA).

**Figure 4:**
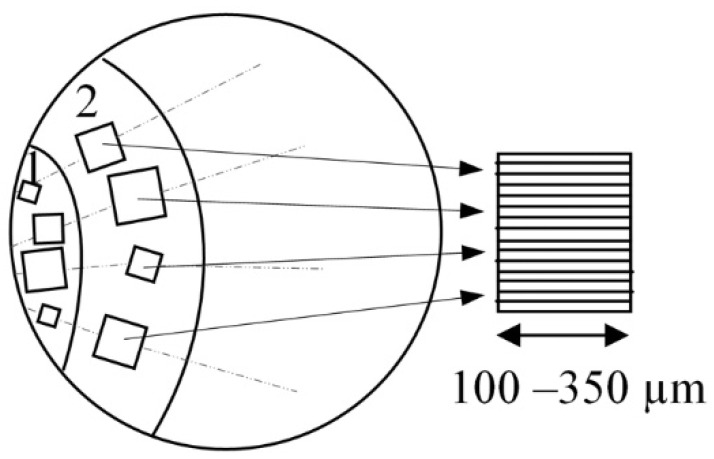
Scanning diagram of the fatigue damage zone: 1—crack initiation zone; 2—crack propagation zone.

Two characteristic zones were revealed on the sample surface: 1—crack initiation zone (around the fracture area) and 2—crack propagation zone (Figure 4). Surface profiles, radial to the boundary interface between zones 1 and 2, were analyzed. In all, 10–13 profiles were obtained within each selected “window” of the zone, providing a data representation of the surface topography induced by defects, with a vertical resolution of ~0.1 nm and a horizontal resolution of ~0.5 µm.

To determine the minimal (critical) scale *l_sc_*, the method for determining the Hurst exponent was applied, which was used by researchers [48,49,50] to study the scale-invariant patterns of structure evolution caused by structural defects. According to the one-dimension profiles, the surface fracture topography was calculated using the correlation function *K*(*r*) from the formula [47]:

(1) $$K\left(r\right)={\langle {\left(z(x+r)-z(x)\right)}^{2}\rangle }_{x}^{1/2}\propto \;r^{H},$$

where *K*(*r*) is the average difference between the values of the surface relief heights *z*(*x + r*) and *z*(*x*)*,* in the window of size *r*, and *H* is the Hurst exponent (surface roughness index).

The representation of *K*(*r*) in logarithmic coordinates with respect to (1) allowed us to evaluate the lower boundary of the scaling range *l_sc_*, and the value of the upper boundary, considering it as the characteristic scale of the process zone *L_pz_*, i.e., the area of correlated behavior of multiscale defect structures. The surface profiles were recorded with a magnification of ×2000.

## 3. Results and Discussion

### 3.1. Microstructure of CG and UFG Ti-45Nb Alloy Samples

**Figure 5:**
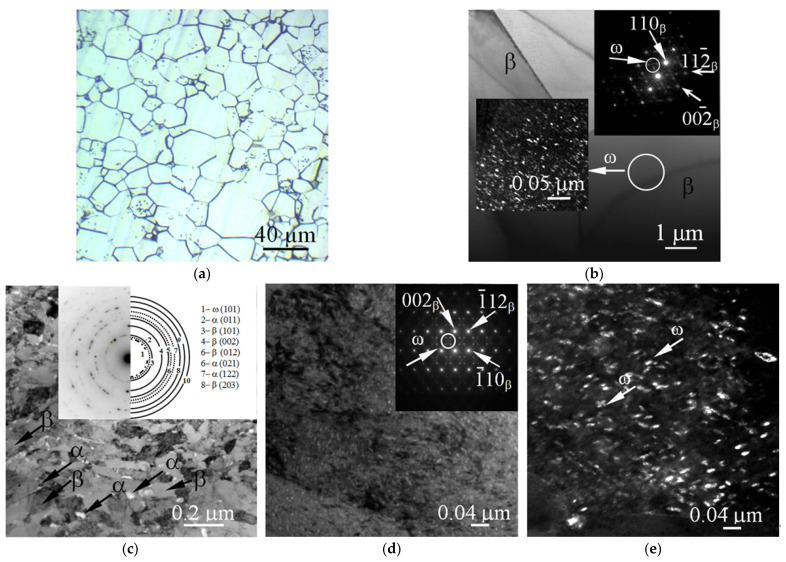
(**a**) Optical micrograph, (**b**–**d**) bright-field TEM micrographs with SAD patterns, and (**b**,**e**) dark-field TEM micrographs of CG (**a**,**b**) and UFG Ti-45Nb samples (**b**–**e**).

The CG and UFG microstructures of the Ti-45Nb samples are shown in Figure 5. After recrystallization annealing, the microstructure of the CG alloy revealed equiaxed β-phase (body-centered cubic (BCC) lattice) matrix grains (Figure 5a,b) with an average size of 45.0 ± 15.0 µm. In the dark-field image, there are ellipsoidal ω-phase (hexagonal primitive lattice) particles of 10 nm in size (Figure 5b, inset) inside the β-phase grains, the volume fraction of which, according to the TEM data, is about 2 vol.%. Dislocation clusters can be observed in the bright-field images. In the UFG alloy, the average sizes of the structure elements (grains, subgrains, fragments) of the β- and α-phase are 0.2 ± 0.1 and 0.05 ± 0.01 µm, respectively.

The TEM bright-field images demonstrate (Figure 5c) β–phase and α-phase subgrains, as well as ω-phase particles in the bulk of β-phase grains (Figure 5d,e). The volume fractions of the α- and ω- phases are 6 and 3 vol.%, correspondingly.

The mechanical properties of the CG and UFG Ti-45Nb samples are listed in Table 2. A detailed description of the microstructure and mechanical properties of Ti-45Nb alloy samples with a different structure subjected to quasi-static stress has been published in [42,43,44].

The UFG structure in the alloy resulted from SPD products increasing the yield strength and ultimate strength by ~2 times. The yield strength of the UFG alloy increased to 620 MPa, while the ultimate strength grew to 1150 MPa. For the CG alloy, σ_0.2_ and σ_B_ are equal to 360 MPa and 630 MPa, respectively. The ultimate deformation before fracture of the UFG alloy is 5.5%. Young’s modulus of the CG alloy is 50 GPa, while that of the UFG alloy increases up to 58 GPa. Thus, the UFG structure of the Ti-45Nb alloy resulting from SPD provides improved mechanical properties and a low Young’s modulus in the range of the elastic moduli that are typical for cortical bones (10–40 GPa).

### 3.2. Gigacycle Loading of the Ti-45Nb Alloy

**Figure 6:**
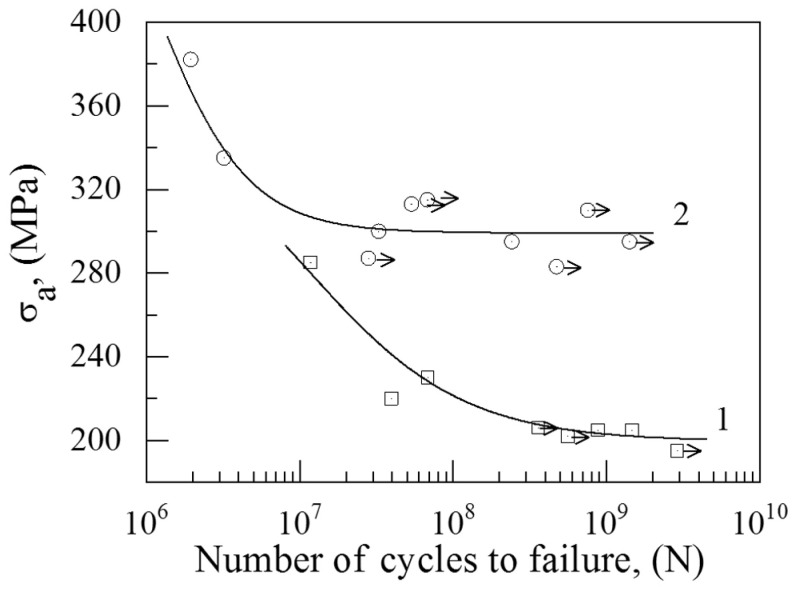
The amplitude of applied stress and fatigue curves of the CG (1) and UFG (2) Ti-45Nb alloys. Arrows indicate those samples which did not fail during the testing process.

The results of the gigacycle fatigue-loading of the CG and UFG Ti-45Nb alloys are depicted in Figure 6. An analysis of the results indicated that the formation of the UFG structure in the alloy resulted in a significant increase of the fatigue strength under gigacycle loading.

The UFG Ti-45Nb alloy samples did not fail after 10^8^ loading cycles, even under an applied stress amplitude of 300 MPa. However, the CG alloy samples failed at a lower stress amplitude of 230 MPa after 10^8^ cycles. The fatigue strength of the UFG Ti-45Nb alloy samples after 10^6^ cycles was 380 MPa, which exceeded the fatigue strength value for the CG alloy (280 MPa) by 1.4 times. It should be noted that the fatigue strength of the UFG alloy samples subjected to 10^9^ cycles was 295 MPa, while that of the CG alloy samples was 195 MPa, which was 1.5 less than the fatigue strength of the UFG alloy. 

Thus, the UFG structure provided a significant increase in fatigue strength compared to the CG structure.

#### Investigation of Fatigue Strength Using the Rizitano–Loung Method

**Figure 7:**
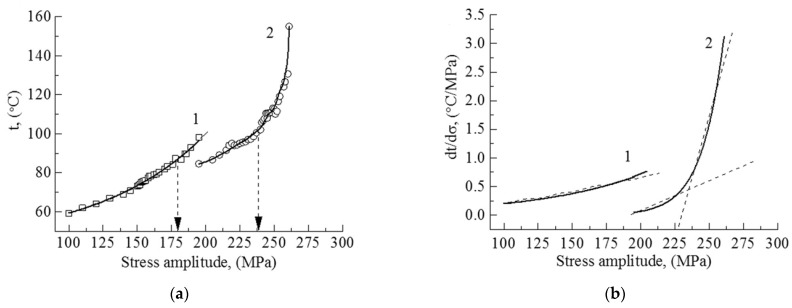
(**a**) The maximal temperature and (**b**) the rate of increasing the maximal temperature on the surface of CG (1) and UFG (2) Ti-45Nb alloys, a function of stress under cyclic loading.

The experimental results for CG and UFG alloy samples, obtained using the Rizitano–Loung method are illustrated in Figure 7. Based on the experimental data, the dependence of maximal temperature on the sample surface on the applied stress amplitude during cyclic loading was plotted. A quintic polynomial function was applied to approximate the dependence. It is evident that during cycling at the chosen stress amplitudes, the maximal temperature for the UFG samples exceeds the temperature for the CG samples. 

According to the Rizitano–Loung method, the inflection point (Figure 7a, indicated by arrows on curves 1 and 2) shows the approximate value of the high-cycle fatigue strength, which is 180 MPa for the CG alloy and 235 MPa for the UFG alloy.

The dependence of the rate of increasing the temperature on the stress amplitude for the alloys is shown in Figure 7b (curves 1, 2). Curve 1 is well approximated by a linear function within a stress amplitude interval of 100–195 MPa for the CG alloy samples, while curve 2 demonstrates two linear segments (195–230 and 240–260 MPa) for the UFG alloy samples.

This two-stage increase of this rate could be associated with the qualitative difference in energy dissipation mechanisms related to the CG and UFG alloy structure [30]. The mechanical energy that is accumulated in the samples is greater for the UFG alloy [30,31,32].

### 3.3. Temperature Field Evolution in Ti-45Nb

**Figure 8:**
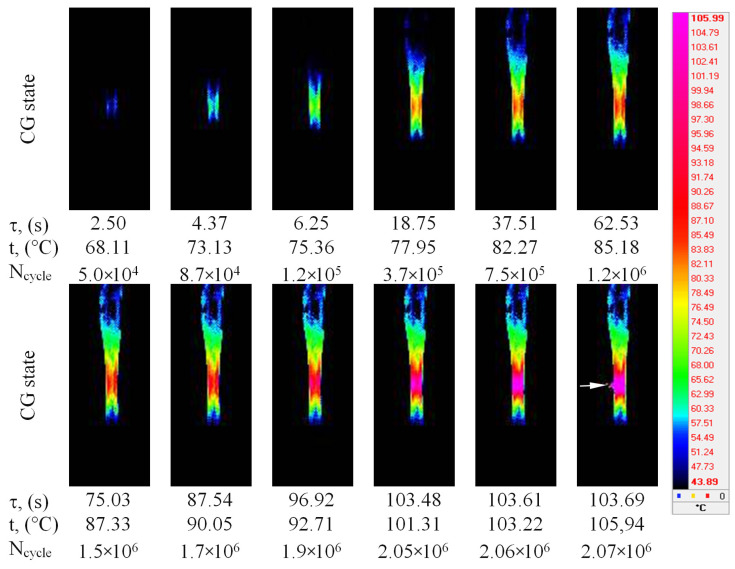
Thermal field images in the infrared zone, recorded during the cyclic loading of the CG Ti-45Nb sample: τ (s)—time, N—number of loading cycles, t—maximal temperature on the surface.

**Figure 9:**
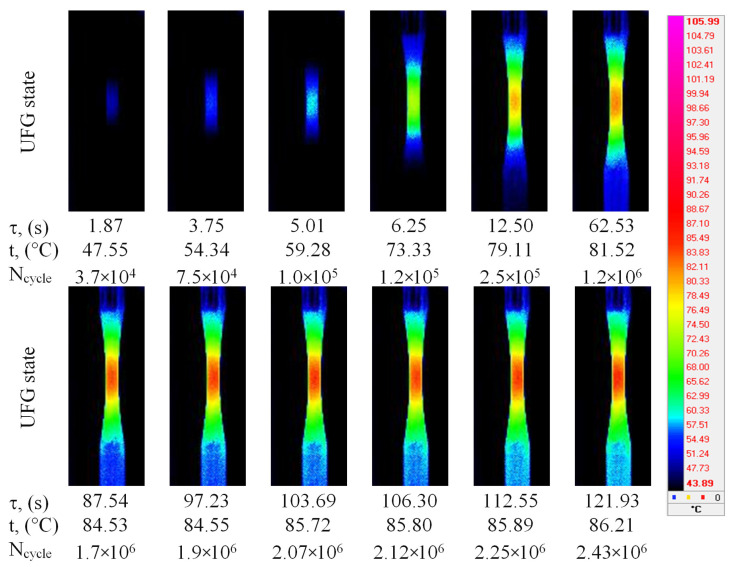
Thermal field images in the infrared zone, during the cyclic loading of UFG Ti-45Nb sample: τ (s)—time, N—number of loading cycles, t—maximal temperature on the surface.

The evolution of thermal fields during the cycle-loading of the Ti-45Nb alloy samples is shown in Figure 8 and Figure 9. Similar behavior can be observed for the two different structures of the Ti-45Nb alloy. Cyclic deformation resulted in the initiation and propagation of the heat-release zone, which area was extended with the increase in testing time. The heat release zone at the maximal temperature is significantly different for the CG and UFG samples (Figure 8 and Figure 9). The area of the thermal field zone was almost the same for the CG and UFG samples after 10^5^ cycles of testing, while qualitative temperature changes appeared after 10^6^ cycles.

The zone of elevated temperature (~105 °C) appeared on the working part of the CG sample and eventually enlarged throughout the entire sample. A temperature jump is observed on the thermal images at the moment of the sample pre-fracture at 2.07 × 10^6^ cycles (indicated by arrows in Figure 8).

The spreading of the temperature field on the surface of the UFG Ti-45Nb alloy was similar to that in the CG alloy. However, temperature stabilization was observed in the middle part of the sample with the increase in testing time. It should be noted that the maximal temperature zone in the surface area of the UFG samples (~86 °C) was less than in the CG samples (Figure 8 and Figure 9). 

**Figure 10:**
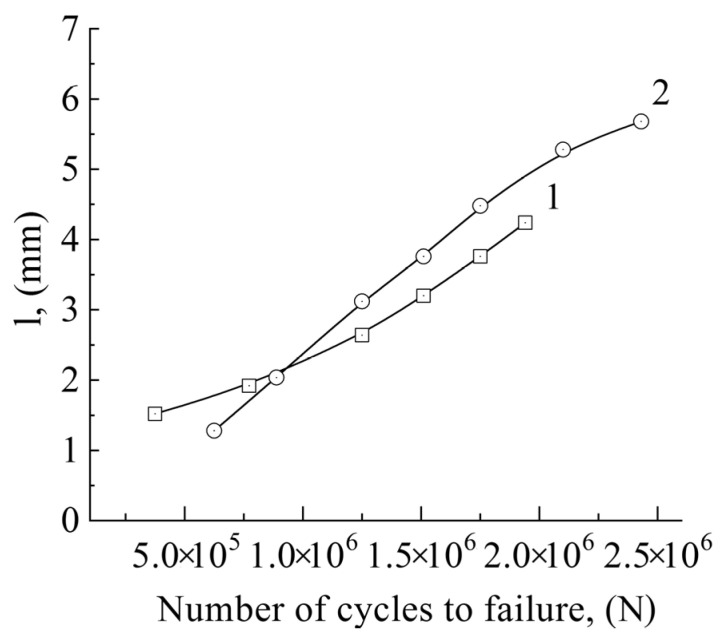
The longitudinal dimension of the heat release zone as a function of the number of loading cycles at the amplitude σ = 195 MPa and the temperature 86 °C; 1—CG sample; 2—UFG sample.

The longitudinal dimension of the thermal zones bounded by isolines corresponding to ~86 °C is depicted for CG and UFG alloys in Figure 10, as a function of the number of loading cycles. It is clearly seen that in the case of the CG alloy, the zone dimension grows nearly linearly with the increasing number of cycles (Figure 10, curve 1). The longitudinal dimension of the heat-release zone changed from 1.5 mm (N = 3.7 × 10^5^) to 4.2 mm (N = 1.9 × 10^6^). Note that in this case, heat losses during interaction with the environment and energy losses during friction in gripping the sample were not taken into account.

The dimension of the heat-release zone in the UFG alloy also increases during the cyclic loading (curve 2) from 1.3 mm (N = 6.2 × 10^5^) to 5.7 mm (N = 2.4 × 10^6^). At the same time, the curve slope for the UFG sample is higher than that for the CG sample. 

Thus, based on the data presented in Figure 10, it can be concluded that heat generated during the cyclic loading of the UFG alloy is more effectively dissipated and involves a substantially larger sample volume than in the case of the CG alloy.

**Figure 11:**
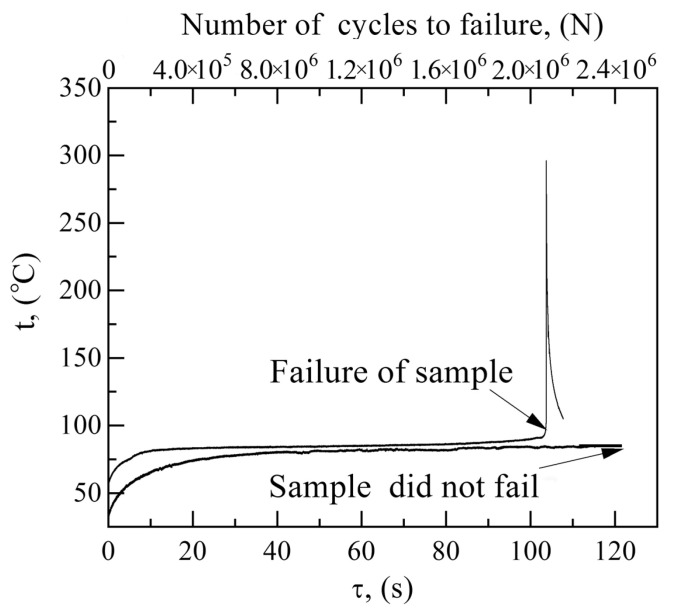
Maximal temperature change in the central area of the CG (1) and UFG (2) samples caused by cyclic loading (loading amplitude σ = 195 MPa) at room temperature.

The maximal surface temperature within the working part of the samples as a function of loading time (loading amplitude σ = 195 MPa) for the UFG and CG Ti-45Nb is illustrated in Figure 11. The CG samples were heated during cyclic testing to 73 °C for 6 s. After cycling for 20 s, the temperature stabilizes in the range of 85–90 °C. During this period, damage to the sample occurred within the multi-cycle zone (10^6^ cycles). The maximal heating was ~105 °C for 103 s. Before the damage occurred, a sharp temperature peak (300 °C) was observed in the CG sample, which could be related to increasing the heat release in the micro-volumes subjected to micro-deformation during fatigue failure. Consequently, an on-site temperature increase could decrease the material strength in micro-volumes, facilitating the formation of new plastic displacements. In this case, the temperature could be significantly higher in local shear bands, where energy dissipation occurs, than in the entire sample.

The temporal evolution of the maximal temperature is the same for UFG and CG Ti-45Nb alloys (before damage). During cyclic loading, temperature increases slightly more slowly in the UFG alloy than in the CG alloy. The UFG samples were heated to 80 °C after 37 s of cycle testing, while the temperature was stabilized at 80–90 °C after 40 s of testing, which corresponds to the 10^6^ cycles. Temperature stabilization after 10^6^ cycles was also observed in the CG samples. It should be noted that, in contrast to the CG samples, the UFG samples did not fail after 2 × 10^6^ cycles at a stress of 195 MPa; the testing was terminated after temperature stabilization at 90 °C. The fact that, in the case of a stabilized temperature, the samples did not fail after 10^6^ cycles indicates that the UFG material enhanced the formation of equilibrium grain-boundary defect systems [30].

Accordingly, qualitative changes in energy dissipation were observed during the cyclic loading of the UFG and CG Ti-45Nb alloys. It has been proved elsewhere [51,52] that CG and UFG aluminum alloys subjected to dynamic compression are characterized by different energy dissipation, which is associated with their different grain sizes and defect micro-structures.

This energy dissipation could be influenced by either characteristic structure features (grain size, defect structure, presence of grain-boundary defects) or phase state (the presence of second-phase dispersion particles). It should be noted that the dispersion-strengthened ω-phase [43] was observed in the CG and UFG Ti-45Nb alloy samples.

The features of the mechanical and thermodynamic reactions in CG and UFG materials are usually associated with a qualitative difference in the collective behavior of an ensemble of dislocations that exhibit signs of critical behavior (structural-scaling transitions). For CC materials, this is accompanied by the formation of ordered dislocation ensembles and the localization of plastic deformation, when the threshold values of stresses (flow stresses) are reached. The behavior of the UFG materials is traditionally associated with a violation of the Hall–Petch law, which does not lead to the formation of ordered dislocation ensembles of dislocations or the localization of plastic deformation and is characterized by an increase in the contribution of stored energy. The formation of the ordered ensembles and the associated effects of the localization of plastic deformation are accompanied by an increase in the dissipative component, which is confirmed by the experiments; this allowed us to conclude that the dissipative abilities of the CG and UFG alloys are different.

### 3.4. Fractal Analysis of the Fracture Surface

Due to a gigacycle fatigue of 10^8^ cycles or more, the damaged sample surface exhibits a clear visible fracture zone, a so-called “fish-eye”, where the localized damaged area is surrounded by fragmented structures [50]. The damage during fatigue testing is related to micro-plastic deformations [39,40,53,54] under cycle-loading conditions, which are initiated by different micro-structure mechanisms. A specific type of localization of fatigue (cyclic) deformation was observed for plastic metals, which could result in crack initiation in the subsurface zone. Based on the surface morphology analysis of damaged areas, cracks were revealed in the subsurface layer that were concentrated at inner defects, such as inclusions, pores and interfacial boundaries. 

**Figure 12:**
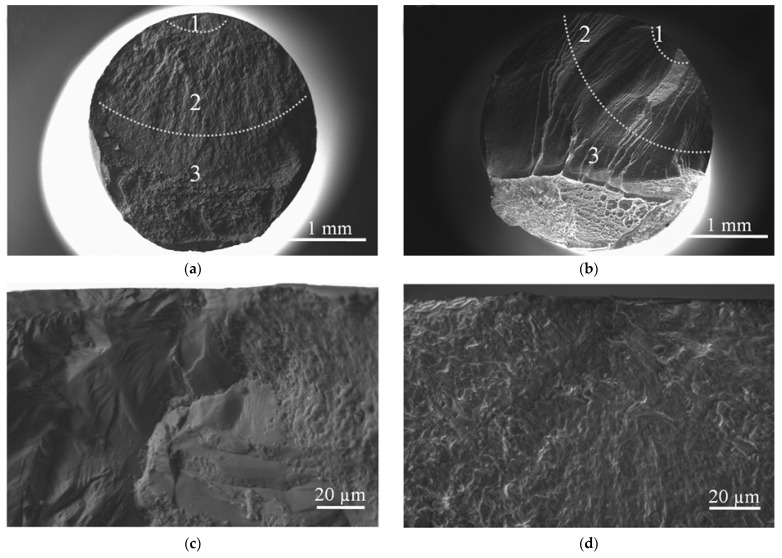

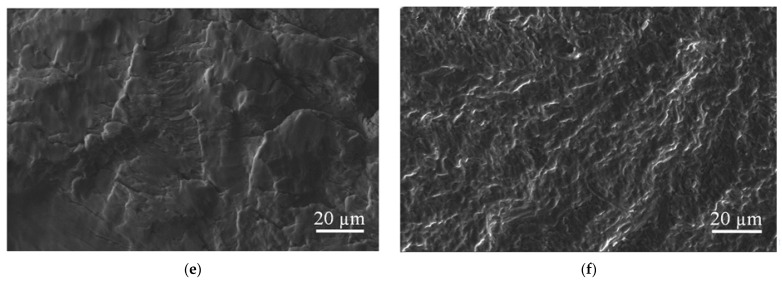
SEM micrographs of fracture surface of (**a**,**c**,**e**) CG and (**b**,**d**,**f**) UFG Ti-45Nb alloy samples: (**b**,**d**) crack initiation zone; (**e**,**f**) crack propagation zone.

Scanning electron microscope (SEM) micrographs of the fracture surfaces of both Ti-45Nb alloy samples are shown in Figure 12. 

Three typical zones were observed on the fracture surface: 1—crack initiation zone; 2—crack propagation and growth zone; 3—rupture zone. Fatigue cracks were generated close to the lateral surface and expanded inward in the samples. The CG samples showed a rough surface (Figure 12a). Rough surface topography, emerging as a faceted surface with small cracks, was observed in the initiation zone (Figure 12c). This zone is distinguished by an area of dimpled microrelief. There are numerous pores and fatigue marks on the fracture surface. Secondary cracks, significant areas of fatigue marks, numerous small pores and dimples appeared in the zone of crack propagation (Figure 12e). The average dimple size in the crack initiation and propagation zones ranged from 4.2 to 8.7 µm.

Compared to the above-mentioned CG samples, the UFG samples showed distinct features on the fracture surface, i.e., lower levels of roughness and small cracks (Figure 12b). The crack initiation zone contains defect structures, such as cracks and fatigue marks coupled with pores and dimples (Figure 12d). The fatigue marks located perpendicular to the crack propagation direction are typical for fatigue failure. The crack propagation zone (Figure 12f) showed a multi-component structure consisting of secondary cracks, pores and dimples. An average dimple size for the crack initiation and propagation zones varied between 5.7 and 9.5 µm.

**Figure 13:**
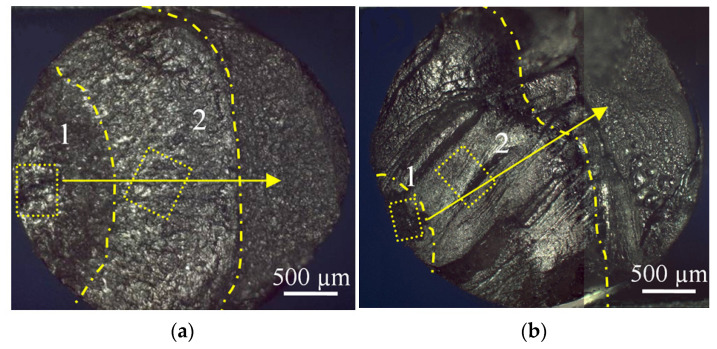
Optical micrographs of the fatigue fracture surface close to the initiation area (zone 1) and within the crack propagation zone (2) of (**a**) UFG and (**b**) CG Ti-45Nb alloy. The rectangles shown by dot lines indicate the selected areas for surface scanning. The solid arrows point the profile measurement direction, corresponding to fatigue crack distribution. The dash dot lines show boundaries of zones 1 and 2.

To investigate the fatigue behavior of the UFG and CG samples during the gigacycle testing, numerical Hurst exponent analysis was performed according to the procedure described in [48,49]. Surface profiles ensured the visualization of fracture relief in different surface zones, both close to and far from the initiation (zone 1) along the main direction of crack propagation (zone 2) in the Ti-45Nb samples (Figure 13a,b).

**Figure 14:**
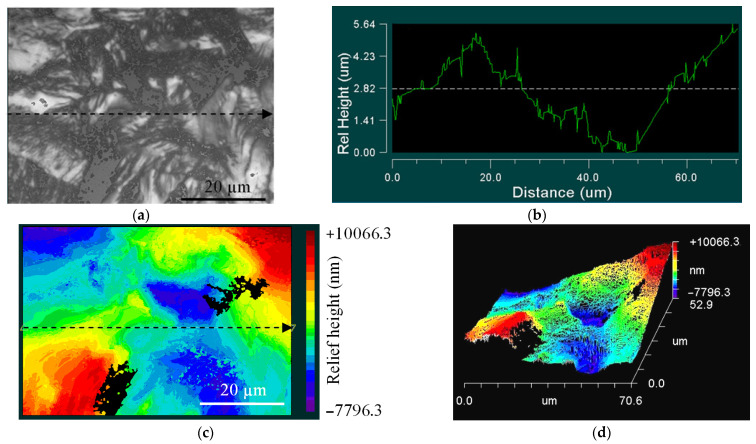
Images of damage to the surface, close to the crack initiation zone (zone 1) of the CG Ti−45Nb alloy: (**a**) optical image of scanning area; (**b**) surface profile; (**c**) 2D-relief map; (**d**) 3D-relief map.

**Figure 15:**
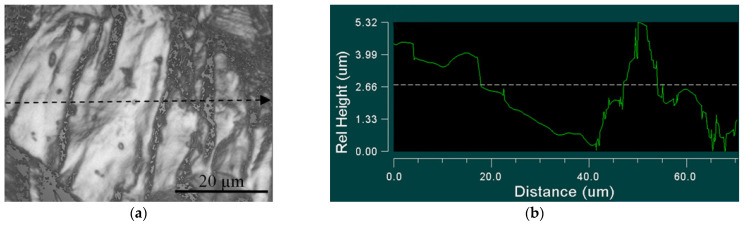

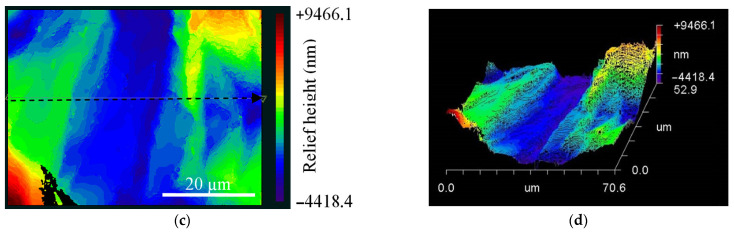
Images of the damage surface in the crack propagation area (zone 2) of the CG Ti-45Nb alloy: (**a**) optical image of scanning area; (**b**) surface profile; (**c**) 2D-relief map; (**d**) 3D-relief map.

Figure 14 and Figure 15 show typical maps revealing the location of the CG sample relief, close to the initiation area (zone 1) and crack propagation area (zone 2). Zone 1 (Figure 14a) is related to the defect accumulation area, and the initiation of fatigue cracks revealed different relief heights (Figure 14b–d).

The difference in the surface topography remains far from the damaged area in zone 2 (Figure 15a). However, the surface was characterized by lesser roughness (Figure 15b–d), compared to zone 1.

**Figure 16:**
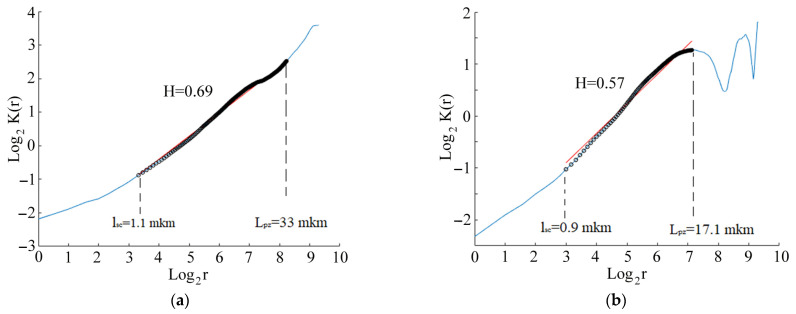
log_2_K(r) as a function of log_2_(r), Hurst exponent coefficient *H* and scales *L_pz_* and *l_sc_* of the CG Ti45Nb alloy sample for different zones: (**a**) zone 1; (**b**) zone 2.

Correlation function *K*(*r*) plotted in logarithmic coordinates for surface profiles in zones 1 and 2 of the CG alloy is shown in Figure 16a,b. The function *K*(*r*) for zone 1 and zone 2 embraced a linear section (Figure 16, full line) within the critical scale *l_s_*_c_ and *L_pz_*. 

The value of the linear section of the correlation function corresponds to the value of the fractal dimension of the crack profile, propagating according to Paris’s law. Both the critical scale *l_sc_* and the area of defect structure *L_pz_* in the crack initiation zone of the CG samples (Figure 16a) increased, while the Hurst exponent remained constant within the range of 1.1–33 µm of the scale parameters. The Hurst exponent coefficient was estimated as 0.69. A significant decrease in the spatial scale interval was revealed in the crack propagation zone (Figure 16b), where the Hurst exponent (H = 0.57) remained constant within the range of 0.9–17.1 µm of the scale parameters. 

**Figure 17:**
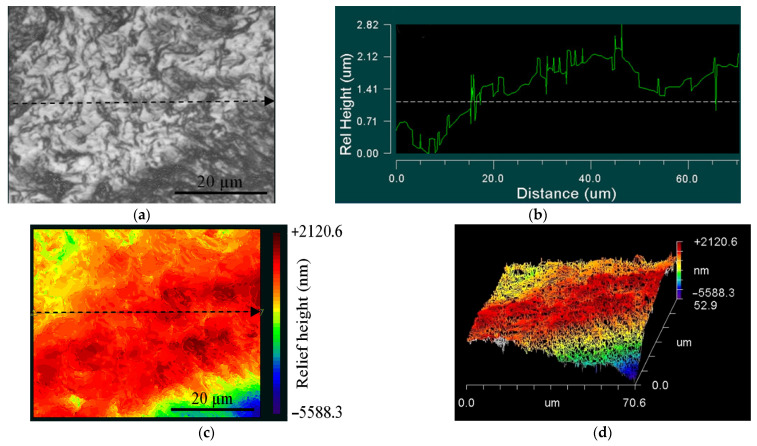
Image of fracture surface close to crack initiation area (zone 1) of UFG Ti-45Nb alloy: (**a**) optical image of scanning area; (**b**) one-dimensional relief map; (**c**) 2D-relief map; (**d**) 3D-relief map.

**Figure 18:**
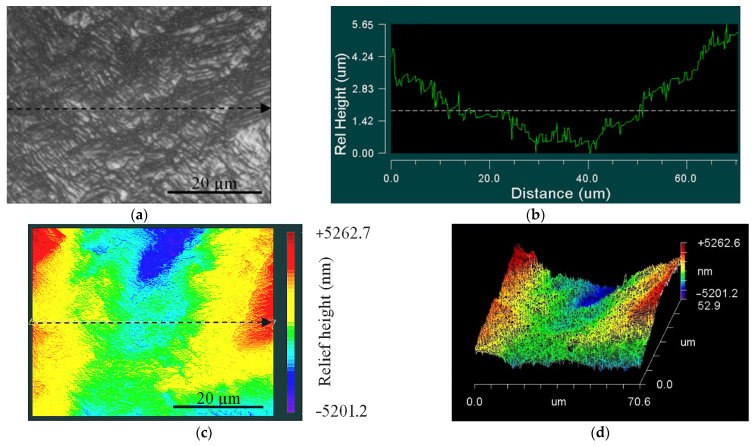
Image of fracture surface in crack propagation area (zone 2) of UFG Ti-45Nb alloy: (**a**) optical image of scanning area; (**b**) one-dimension relief map; (**c**) 2D-relief map; (**d**) 3D-relief map.

Typical relief maps of the UFG alloy samples, plotted for zone 1 and zone 2, are illustrated in Figure 17 and Figure 18.

Areas of different relief heights were observed in both zone 1 (Figure 17a) and zone 2 (Figure 18a) for the UFG and CG samples. Zone 1 (surrounding the crack initiation area), where defect accumulation and crack fatigue initiation originated (Figure 17b–d), has significantly higher roughness compared to zone 2 (Figure 18b–d).

**Figure 19:**
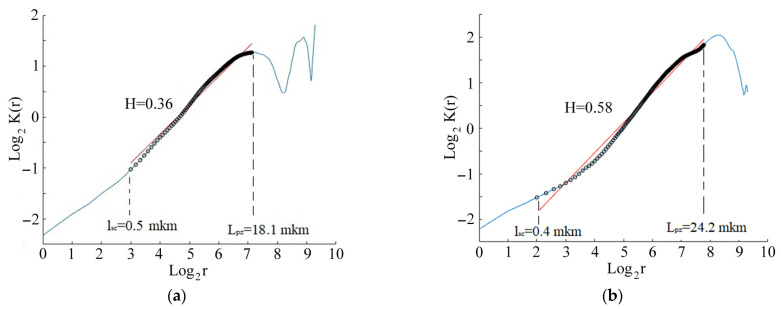
Dependence of log_2_*K*(*r*) on log_2_(*r*), Hurst exponent H and scales *L_pz_* and *l_sc_* of the UFG Ti45Nb alloy sample for (**a**) zone 1; (**b**) zone 2.

The correlation function *K*(*r*), plotted in logarithmic coordinates for the surface profile of the UFG alloys, is illustrated in Figure 19. The function *K*(*r*) for zones 1 and 2 shows the same linear segment for the UFG and CG alloys. The slope of the linear segment corresponds to the fractal dimension of the crack profile, according to Paris’s law. There is a significant decrease in the spatial scale interval for the UFG samples (Figure 19a,b), where the Hurst exponent remained constant for the two zones, within the range of 0.4–24.2 µm.

The average Hurst exponent and critical scales *L_pz_* and *l_sc_* in zone 1 and zone 2 for CG and UFG Ti-45Nb alloy are illustrated in Table 3.

Thus, scale invariance, the Hurst exponent, and their related scales, indicating fatigue failure development, were determined based on the numerical analysis for the fracture surface of the CG and UFG Ti-45Nb alloy samples, which revealed the surface areas of the crack generation and initiation formed in the gigacycle regime.

## 4. Conclusions

The conclusions from the present research are as follows.

1. The dependence of the UFG Ti-45Nb alloy on fatigue limit was determined in different cyclic loading regimes. It was proved that the formation of the UFG Ti-45Nb alloy samples by multidirectional forging (abc–forging) and further rolling resulted in increasing the fatigue limit in the gigacycle regime up to 295 MPa, which is 1.5 times more compared to the CG sample (fatigue limit—195 MPa).
2. The fatigue limit under multi-cyclic loading for the Ti-45Nb alloy samples was estimated by applying the Rizitano–Loung method. The fatigue limit for the CG alloy sample was 180 MPa, while it was 235 MPa for the UFG sample.
3. During the cyclic loading, the longitudinal size of the heat source at a given temperature increases, and the rate of growth of the longitudinal size of the heat source for the alloy in the UFG state is higher than for the CG alloy samples.
4. Characteristic deformation zones were revealed in the UFG Ti-45Nb alloys that were subjected to cyclic loading. Scale invariance (the Hurst exponent) and their related scales, indicating fatigue damage development, were determined. This made it possible to reveal the initiation and propagation of cracks on the surface-damage area in the gigacycle fatigue regime. Both the critical scale *l_sc_* and area of defect structure *L*_pz_ in the crack initiation zone of the CG sample increased, while the Hurst exponent remained constant within the range of (1.1–32.9) µm of the scale parameters. A significant decrease of the spatial-scale interval was revealed in the crack propagation zone, where the Hurst exponent (H = 0.57) remained constant within the range of (0.9–17.1) µm. The UFG sample demonstrated a significant decrease in the spatial scale range, where the Hurst exponent remained constant in the two zones, i.e., in the interval of 0.4–24.2 µm.

## Figures and Tables

**Table 1:** Chemical composition of Ti-45Nb alloy.

Element Content, wt.%					
Ti	Nb	Cr	Fe	C	W
55.3	44.3	≤0.1	≤0.06	≤0.05	≤0.1

**Table 2:** Mechanical properties of Ti-45Nb alloy [43].

Alloy Structure State	σ_0.2_, MPa	σ_B_, MPa	ε, %	E, GPa	d_cp_, µm
CG	360 ± 10	630 ± 10	16.0	50	45.0 ± 15.0
UFG	620 ± 10	1150 ± 10	5.5	59	0.2 ± 0.1

σ_0.2_—yield strength; σ_B_—ultimate strength; ε—ultimate plastic deformation before fracture; H_μ_—microhardness, E—Young modulus (experimentally determined from the three-point bending experiments), d_cp_—average structure element size.

**Table 3:** Average Hurst exponent and critical scale *L_pz_* and *l_sc_* for the UFG and CG Ti-45Nb alloys.

Stress Amplitude σ, MPa	Number of Cycles, N	Zone	Hurst Exponent, H	Critical Scales *l_sc_*, μm	Critical Scales *L_pz_*, μm
CG state					
205	5.6 × 10^8^	1	0.69 ± 0.01	1.1 ± 0.4	32.9 ± 3.4
		2	0.57 ± 0.02	0.9 ± 0.5	17.1 ± 2.4
UFG state					
295	2.4 × 10^8^	1	0.36 ± 0.02	0.5 ± 0.2	18.1 ± 2.4
		2	0.58 ± 0.04	0.4 ± 0.1	24.2 ± 2.7
